# An fMRI Based Study of the Neural Correlates of OCD Sub-Types

**DOI:** 10.1192/bjo.2024.118

**Published:** 2024-08-01

**Authors:** Shagun Chahal, Pratap Sharan, Senthil Kumaran, Gagan Hans

**Affiliations:** 1 GMMH, Manchester, United Kingdom; 2 AIIMS, New Delhi, India

## Abstract

**Aims:**

1. To study the neural correlates of OCD using functional MRI.
2. To compare the neural correlates of the pure washer dimension of OCD with other dimensions of OCD and healthy controls.

**Methods:**

It was a cross-sectional, case-control study conducted from 2018 to 2021. OCD patients were recruited with purposive sampling from outpatient attendance at All India Institute of Medical Sciences, New Delhi following the inclusion and exclusion criteria. The patients were divided into two groups i.e. washing sub-type and non-washing/other sub-type based on dimensional YBOCS score. The healthy control group consisted of age and sex-matched healthy individuals. Each group had 10 individuals. The participants underwent functional MRI with resting fMRI and activation task-based MRI. Activation tasks included a cognitive task i.e. Stroop test and an affective task which included trigger words for OCD tailored according to the patient's triggers for OCD.

The results were studied for significance within a group and also compared among the three groups and between OCD patients and healthy controls as well.

**Results:**

In OCD-specific task using trigger words, the right frontal gyrus, right medial frontal gyrus, and left cingulate gyrus showed hyperactivation in the washer OCD subtype group. After correction for family-wise error, p-FWE (<0.05) corrected < 0.05, there was so significant result. The non-washing subtype had no significant areas of activity on the OCD specific task.

But the combined OCD patient group (compared with controls), had hypoactivation of the right inferior frontal gyrus and fusiform gyrus at p-unc (<0.001) in the OCD task.

In the Incongruent part of the Stroop task, the non-washer subtype had hypoactivation of the right caudate body compared with healthy controls at p-FWE (<0.05).

In the congruent Stroop task, washer OCD subtype, the right insula was found to be hyperactive at p-FWE (<0.05).

**Conclusion:**

Previous studies comparing activation on cognitive tasks in OCD patients and healthy controls have revealed differences in CSTC circuits as well as cerebellum and parietal areas. The washing symptom dimension is associated with insular hyperactivity in both emotional and cognitive tasks. It is associated with stimuli related to disgust. The role of the insula is being researched in functions like attention and response inhibition. Our study, with all its limitations, could replicate the insular findings in washing-subtype of OCD. With a better sample size, we may be able to explore further the findings that have not attained levels of significance in our study.

